# Examination of social network members’ influence on daily drinking: A pilot study

**DOI:** 10.1016/j.abrep.2026.100681

**Published:** 2026-02-20

**Authors:** Matthew K Meisel, Alexander W. Sokolovsky, Kristina M. Jackson, Shannon R. Forkus, Nancy P. Barnett

**Affiliations:** a Center for Alcohol and Addiction Studies, School of Public Health, Brown University Providence, RI 02903, USA; b Department of Management, Policy, and Community Health, School of Public Health, University of Texas Health Science Center at Houston, Houston, TX, USA; c Rutgers Addiction Research Center and Robert Wood Johnson Medical School, Rutgers University, Piscataway, NJ, USA

**Keywords:** Social networks, Alcohol use, Daily diary, Pilot study

## Abstract

**Objective:**

Research on young adult alcohol use often overlooks the influence of specific social network members on daily alcohol use. This pilot study combined egocentric social network methods with a daily diary design to examine how network members influence drinking at the day level.

**Methods:**

Participants (N = 21) identified six social network members they frequently drank with and saw in person and then reported on these individuals in a 21-day study. Daily reports captured self-reported alcohol use and social network members presence and alcohol use from the previous day.

**Results:**

Across 417 morning reports, participants drank on 77 days (18.5%), consuming an average of 2.36 drinks (*SD* = 1.70) on those days. Linear mixed-effects models showed that being with a given network member who was drinking was associated with consuming 1.75 more drinks than the participant’s average. Network members who contributed to higher alcohol use were more likely to be people the participant intended to drink with in the future and who had frequently consumed alcohol in the past month, regardless of whether it was with the participant.

**Conclusions:**

The findings from this pilot study provide preliminary evidence that the drinking of and anticipating future drinking with certain network members contributes to greater alcohol use and suggests that interventions could use personalized feedback to help individuals recognize the network members who facilitate heavier drinking.

## Highlights

• Daily drinks increased with each additional network member who was drinking.
• Being with a drinking network member increase daily use by nearly two drinks.
• Network members who were frequent drinkers were influential.
• Network members who participants expected to drink with were influential.

Daily diary studies with young adults have found that social context is the strongest predictor of alcohol initiation and consumption at the day level (O'Donnell et al., 2019). Individuals are more likely to consume alcohol and in greater quantities on days when they are with others who are drinking (O'Donnell et al., 2019). Moreover, multiple studies demonstrate that descriptive norms are positively associated with alcohol consumption at the event or day level (Cullum et al., 2012, Hamilton et al., 2021, Lewis et al., 2020, O’Grady et al., 2011). One limitation of this line of research is that using aggregate perceptions of peer use (e.g., “how often do your friends drink?”) assumes all friends are equally influential, which does not reflect the real-world dynamics of interpersonal influence.

Social network analysis (SNA) provides a framework to examine how specific network members influence behavior (Borgatti et al., 2009). Prior SNA studies indicate that other drinkers and “drinking buddies” appear to exert the greatest influence on one’s alcohol use (Knox et al., 2019, Lau-Barraco et al., 2012, Lau-Barraco and Linden, 2014, Meisel et al., 2015, Reid and Carey, 2018); however, these studies did not utilize a within-person (e.g., daily diary) design which allows for the examination of changes within individuals over time. Only one study, to date, has merged a daily diary design and SNA to examine alcohol use. Bragard et al. (2024) found that individuals were 2.11 times more likely to drink when they were exposed to one additional drinking peer compared to their average level of peer network exposure. However, the presence of an additional drinking peer did not significantly influence the amount of alcohol individuals consumed after adjusting for network total drinks. They also found that individuals were 1.13 times more likely to drink and consumed 0.15 more drinks when their network consumed greater amounts of alcohol than usual. Bragard et al. (2024) examined relative exposure, assessing how deviations from an individual's typical level of drinking network members’ exposure influenced their own drinking. Our study extends this work by examining absolute exposure, directly linking the number of drinking network members present at a given moment to increases in alcohol consumption. This approach offers a more concrete understanding of how the drinking of network members influences one’s own drinking.

The current study was designed to evaluate the initial utility of integrating daily longitudinal data with social network assessments to examine proximal peer influence on alcohol use. Accordingly, the study had two aims. First, it examines how the presence and number of social network members who are drinking contributes to an individual’s drinking quantity on that day. Second, it identifies which types of network members are most influential at the daily level. This investigation leverages data from a study investigating passive assessment of peer presence that had high compliance and reports of daily drinking for each participant (see Barnett et al., 2024, Jackson et al., 2024 for full study protocol).

## 1 Method

### 1.1 Participants and procedure

For this study, a convenience sample of 21 participants was recruited from the Providence, RI community. Inclusion criteria were: (a) ages 18 to 35; (b) able to read English; (c) own an Android smartphone with a data plan; (d) Android OS 11 or later; (e) carry phone throughout the day; and (f) be willing to approach friends to participate. Exclusion criteria were: 1) in treatment or seeking treatment for alcohol or substance use and 2) plans to travel during the “next few months” corresponding to the study period. Participants completed a baseline in-person session, 21 days of repeated daily surveys via the PiLR app (https://pilrhealth.com) developed by MEI, and an exit interview. The study was approved by the Brown University institutional review board, and all participants provided informed consent.

The final sample had a *M* age = 23.57 (*SD* = 4.27) and most identified as a man (57.1%) or a woman (38.1%). Racial breakdown was: 38.1% White, 38.1% Asian, 9.5% Multiracial, 4.8% Black, 4.8% American Indian or Alaska Native, and 4.8% Other. Most of the sample (81.0%) identified as non-Hispanic.

## 2 Measures

### 2.1 Baseline assessment

Participants completed a brief demographic questionnaire and a measure of past-month alcohol use. In an interview-administered social network interview (SNI), participants named six people who were important to them and who were around their age. They were asked to prioritize people they drank with in-person often (if that network member drank) and then those they saw in-person often. Participants answered several questions about each network member (see Table 1).

**Table 1:** Descriptive statistics of the network members who the participant drank with and who were also drinking (n = 50).

Variables	M (SD)	%
*Network demographics*		
Age	23.48 (4.05)	
Gender		
Man		49.0
Woman		44.9
Non-binary		6.1
*Network composition*		
Length of relationship	3.72 (1.70)	
0–3 months		20.0
4–6 months		6.0
7–12 months		6.0
1–2 years		34.0
2–3 years		18.0
3 or more years		16.0
Relationship type		
Friend		84.0
Partner/significant other		12.0
Casual acquaintance/co-worker		2.0
Sibling or cousin		0.0
Other family member		0.0
Other		2.0
Confide in each other	1.74 (1.03)	
Rarely or never		18.0
Seldom		14.0
Often		44.0
Frequently		24.0
Socialize	2.22 (0.71)	
Rarely or never		0.0
Seldom		16.0
Often		46.0
Frequently		38.0
*Network alcohol use*		
Drinking together	1.72 (1.46)	
Drinking buddy		
No		58.0
Sometimes		32.0
Yes		10.0
Envision yourself drinking with this person	2.90 (1.40)	
Definitely not		10.0
Probably not		10.0
Maybe, depends		12.0
Probably yes		16.0
Definitely yes		52.0
Frequency of network member drinking	2.84 (1.06)	

**Note**: The characteristics of the network were self-reported by the participant. Length of relationship was coded on a 6-point scale ranging from *0-3 months* (1) to *3 or more years* (6). Confide in each other and socialize were coded on a 4-point scale ranging from *Rarely or never* (0) to *Frequently* (3). Envision yourself drinking with this person was assessed on a 5-point scale ranging from *Definitely not* (0) to *Definitely yes* (4). Past-month frequency of drinking together and how often the network member drank alcohol were measured on an 8-point scale: ranging from *Not in the past month* (0) to *Daily* (7).

### 2.2 Morning reports

Each morning, participants were presented with a pre-populated list of their friends derived from the SNI and were asked “Yesterday, who were you around for any length of time?” For each network member they indicated being around, participants were asked “Of the people you were with yesterday, who was drinking?” Participants also reported the number of drinks they consumed the previous day.

### 2.3 Data analysis

To evaluate the influence of proximal network member drinking on the alcohol consumption of participants, we structured the data such that each row corresponded to one day for each participant-network member pair (n = 2,508) and limited analyses to days when network members were present (n = 847, 33.8%) to avoid confounding our focal findings with the effect of network member presence.

For Aim 1 analyses focused on the presence of network members who are drinking, we fit a linear mixed-effects model (LMEM) (Gałecki and Burzykowski, 2013, Hedeker, 2005) with an unstructured covariance matrix regressing number of daily drinks consumed onto daily network member drinking. We examined two indices of network member drinking: any drinking and number of drinks. We included a covariate for weekday (versus weekend, i.e., Friday and Saturday). Data were modeled with a 3-level structure such that days were clustered within network members, who were clustered within participants. We disaggregated daily network member drinking into within- and between-subjects components by person-mean centering drinking around the proportion of days a given network member drank (Enders and Tofighi, 2007, Yaremych et al., 2023). We multiplied proportion of drinking days by 10 so the scale would range from 0 to 10. Thus, a one-unit change represents a 0.10 change in the original proportion (10 percentage points). Random intercepts accounted for clustering and a random slope for the focal network member drinking variable accounted for heterogeneity in network member influence. To facilitate the interpretation of the modeled network member drinking influence measure, the random slopes of network member drinking were extracted and plotted against the difference in number of drinks consumed by a participant when that network member was present and drinking from the participant’s own mean number of drinks on drinking days. The number of ‘excess’ drinks associated with the presence of that drinking network member is shown visually for each participant (denoted by study ID). We also explored the interaction between network member drinking and weekday on the outcome of participant drinking; however, this effect was non-significant and thus excluded from our final model.

For Aim 1 analyses focused on the number of proximal network members who are drinking, we restructured the data such that each row corresponded to one day for each participant and aggregated the number of drinking network members who were present on a given day. We fit an LMEM regressing the number of daily drinks consumed onto the number of proximal drinking network members, also including weekday as a covariate and disaggregating between- and within-subjects effects. Data were modeled with a 2-level structure such that days were clustered within participants. A random intercept was included to account for mean differences in drinking at the participant level. These models were fit using *glmmTMB* (Brooks et al., 2017) in R 4.4.1 (Team, 2020).

For Aim 2 analyses, using data from the baseline social network interview and the random slopes for daily drinking, we examined differences in the random slope as a function of network members attributes (i.e., gender, drinking frequency) and relationship characteristics (i.e., length known, relationship type). These analyses were conducted using bivariate correlations, independent samples t-tests, and one-way ANOVA tests. Although we considered using cross-level interactions to directly probe how network member attributes (i.e., L2 moderating variables) were related to index participant drinking, these models were underpowered due to the number of observations, yielding unstable estimates. Random slopes from Aim 1 were thus chosen as outcomes for Aim 2 as these are effectively aggregates across the clustering unit (network member). These analyses were conducted in SPSS 29.0.

## 3 Results

Participants completed 417 morning reports and drank on 18.5% of days (*n* = 77), consuming an average of 2.36 drinks (*SD* = 1.70) on drinking days. The mean number of drinks per day was 0.44 (*SD* = 1.17; range 0-8). Across all days, participants reported being around at least one network member on 78.2% (*n* = 330) and were around *M* = 2.03 (*SD* = 1.74) network members. Across all days, participants were around at least one network member who was drinking on 17.5% days (*n* = 73) and who was drunk on 4.5% days (*n* = 19).

**Fig. 1:**
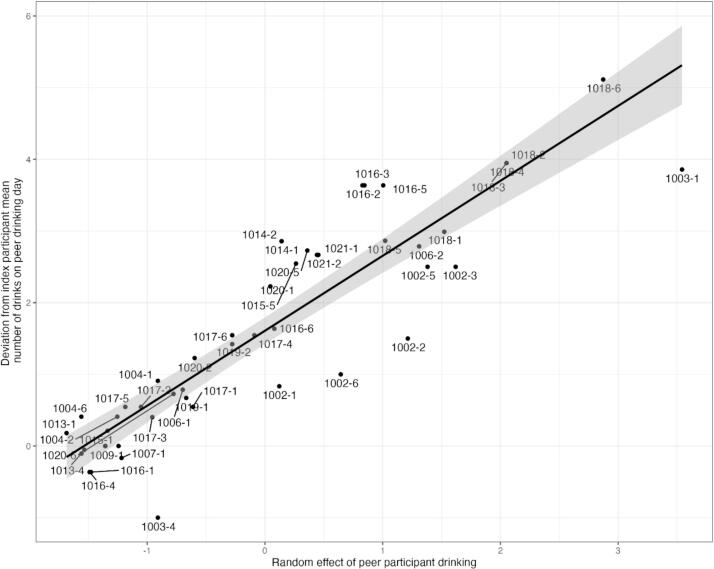
Relationship between network member drinking influence (random slope deviation from fixed effect) and ‘excess’ drinks consumed by participants versus own mean drinking. The x-axis is the random effect of peer participant drinking with 0 being the mean across participants. The y-axis is the mean number of drinks when that network member is around versus that participant’s mean number of drinks. Any network member that is above 0 on the y-axis exerts a positive influence on participants’ number of drinks. Note: Each data point represents a network member with the participant ID number followed by the network member ID number.

In the LMEM for Aim 1, we found that being around a network member who was drinking (versus a network member who was not drinking) was associated with consuming 1.75 more drinks on that day (CI: 1.36-2.13; *p* < .001, i.e., within-subjects effect; see Fig. 1). Further, being around network members who drank on a greater proportion of days they were with the participant was associated with consuming 0.18 more drinks per 10% (0.1) increase in the proportion of drinking days (CI: 0.14-0.22; *p* < .001, i.e., between-subjects effect; see Supplemental Table 1 for full model results). For example, a participant whose network members drank 50% out of the network-member-present days, is predicted to drink 0.36 drinks more (on a typical day) than a participant whose network members drank on only 30% out of the network-member-present days. Results from the LMEM regressing number of drinks onto the number of proximal drinking network members, controlling for weekdays, found that for each additional proximal drinking network member in a given day, the predicted number of drinks consumed by participants on that day increased by 0.89 (CI: 0.75-1.02; *p* < .001, i.e., within-subjects effect; see Supplemental Table 2 for full model results). Further, participants with more proximal drinking network members on average consumed 0.85 more drinks per member (CI: 0.25-1.46, *p* = .007, i.e., between-subjects effect).

The dataset for the Aim 2 analysis consisted of participant-provided information about 50 network members who participants drank with and who also were drinking. Bivariate correlations indicated that there was a significant association between the network member’s impact on participant drinking and the following: (1) the participant’s perception that they would drink with this network member in the future (*r*(50) = .39, *p* = .005), and (2) the participant’s perception of how often the network member drank, with or without them, in the past month (*r*(50) = .35, *p* = .013). All other associations were non-significant (See Supplemental Table 3).

## 4 Discussion

The current study was designed as a pilot to evaluate the utility of integrating intensive daily-level longitudinal data with social network assessments to better understand proximal peer influence on alcohol use. We demonstrated that the specific drinking behavior of a single network member influences alcohol use at the day-level, consistent with prior work integrating daily methods and network analysis (Bragard et al., 2024). Specifically, we found that participants consumed more alcohol on days when they were around (any) network members who were drinking, and that drinking quantity increased as the number of drinking network members present increased. Our second main finding was that network members who contributed to greater drinking than average were more likely to be someone that the person had envisioned drinking with in the future and who drank frequently, with or without the participant, in the past month. We can assume then that network members who contribute to greater drinking are engaging in specific behaviors towards an individual that contribute to greater alcohol use. It might also be that individuals intentionally seek out frequent drinkers to socialize with when they want to drink, knowing those friends will facilitate their behavior. This may be because participants regularly drink with the person and/or may have concrete plans to drink with them in the future. Together, these findings demonstrate the utility of combining network analysis and daily diary methods for identifying which network members are most influential.

Based on these preliminary findings, we offer several suggestions for future research. First, network studies of peer influence on alcohol use would benefit from moving toward real-time assessments (e.g., ecological momentary assessment) to further understand social influence processes. Second, in the network assessment, participants were instructed to nominate peers who they drank with in person often and those they saw in person often. There are multiple ways to identify social network members; future network assessments may benefit from prioritizing individuals who are perceived to drink frequently and with whom participants anticipate drinking with, as these network members are likely to exert the strongest influence on alcohol use. Third, while our study focused on network members who contributed to greater alcohol risk, there may be network members who are more protective, such that they contribute to less alcohol use (Meisel & Barnett, 2017). Being around these network members (such as non-drinking or light drinking friends and significant others) may actually be protective of greater alcohol use. Lastly, a larger, fully powered trial is needed to confirm these preliminary findings. Future studies could also benefit from restricting eligibility to only those who reported recent drinking, consistent with other studies (e.g., Bragard et al., 2024).

## 5 Author Note

This research was supported in part by grant numbers K01AA025994 (PI Meisel), K08DA048137 (PI Sokolovsky) and R21AA027329 (MPI Barnett and Jackson). NIH had no role in the study design, collection, analysis, or interpretation of the data, writing the manuscript, or the decision to submit the paper for publication.

## CRediT authorship contribution statement

**Matthew K Meisel:** Writing – review & editing, Writing – original draft, Formal analysis, Conceptualization. **Alexander W. Sokolovsky:** Writing – review & editing, Writing – original draft, Formal analysis. **Kristina M. Jackson:** Writing – review & editing, Funding acquisition. **Shannon R. Forkus:** Writing – review & editing, Conceptualization. **Nancy P. Barnett:** Writing – review & editing, Funding acquisition.

## Declaration of competing interest

The authors declare that they have no known competing financial interests or personal relationships that could have appeared to influence the work reported in this paper.

## Data Availability

Data will be made available on request.
