# Supplementary material for: Examination of social network members’ influence on daily drinking: A pilot study
Source: Addict Behav Rep. 2026 Feb 20;23:100681. doi: 10.1016/j.abrep.2026.100681 (PMC12963908; doi:10.1016/j.abrep.2026.100681)
Supplement: Supplementary Data 1 [file mmc1.docx]

**Supplemental Table 1**. Fixed and random effects regressing participant number of drinks onto disaggregated network member (any) drinking on that day (WS) and proportion of network member drinking days, aggregated across all study days (BS) on days when network members are proximal to the participant

|  | |  | **95% CI** | |  |
| --- | --- | --- | --- | --- | --- |
| *Fixed effects* | | **Estimate** | **Low** | **High** | ***P*** |
| *(Intercept)* | | 0.63 | 0.47 | 0.79 | <.001 |
| Network member drinking  (*WS* *deviation from BS proportion*) | | 1.75 | 1.36 | 2.13 | <.001 |
| Network member proportion drinking days  (*BS mean, unit = 10% increase)* | | 0.18 | 0.14 | 0.22 | <.001 |
| Weekday (ref: Weekend) | | -0.55 | -0.68 | -0.43 | <.001 |
| *Random effects* | |  |  |  |  |
| \| σ^2^ \| 0.59 \| \| --- \| --- \| \| τ_00_ _friend:id_ \| 0.21 \| \| τ_11_ _friend:id.drink_dev_ \| 1.60 \| \| ρ_01_ _friend:id_ \| 0.90 \| \| ICC \| 0.37 \| \| N _friend_ \| 101 \| \| N _id_ \| 21 \| | |  |  |  |  |
| Marginal R^2^ / Conditional R^2^ | 0.353 / 0.590 | |  |  |  |

**Note**: CI = confidence interval; WS = within-subjects; BS = between-subjects. Friend = network member.

**Supplemental Table 2**. Fixed and random effects regressing number of drinks onto disaggregated number of proximal network members who are drinking that day (WS) and mean proximal network members who are drinking, aggregated across all study days (BS) on days when network members are proximal to the participant

|  | |  | | **95% CI** | |  |
| --- | --- | --- | --- | --- | --- | --- |
| *Fixed effects* | | **Estimate** | | **Low** | **High** | ***P*** |
| *(Intercept)* | | 0.32 | | -0.04 | 0.67 | .078 |
| Number of proximal drinking network members  (*WS deviation from BS participant mean)* | | 0.89 | | 0.75 | 1.02 | <.001 |
| Mean proximal drinking network members  (*BS mean proximal drinking*) | | 0.85 | | 0.23 | 1.46 | .007 |
| Weekday (ref: Weekend) | | -0.21 | | -0.44 | 0.02 | .075 |
| *Random effects* | |  | |  |  |  |
| \| σ^2^ \| 0.81 \| \| --- \| --- \| \| τ_00_ _id_ \| 0.19 \| \| ICC \| 0.19 \| \| N _id_ \| 21 \| | |  | |  |  |  |
| Marginal R^2^ / Conditional R^2^ | 0.371 / 0.490 | |  | |  |  |

**Note**: CI = confidence interval; WS = within-subjects; BS = between-subjects. Friend = network member.

**Supplemental Table 3.** Relationship between the network member’s impact on participant drinking (i.e, the random slope) and network members attributes.

| **Correlations between the network member’s impact on participant drinking and continuous network member attributes** | | | | | | | | |
| --- | --- | --- | --- | --- | --- | --- | --- | --- |
|  |  | Age | Length of relationship | Confide in each other | Socialize | Drinking together | Envision yourself drinking with this person | Frequency of network member drinking |
| Random slope | *r* | 0.179 | 0.23 | 0.164 | 0.186 | 0.049 | 0.391 | 0.347 |
|  | *p value* | 0.213 | 0.107 | 0.254 | 0.197 | 0.735 | 0.005 | 0.013 |
|  |  |  |  |  |  |  |  |  |
| **Does the network member’s impact on participant drinking differ by categorical network member attributes (ANOVA)?** | | | | | | | | |
| Gender identity |  |  |  |  |  |  |  |  |
|  | Sum of Squares | df | Mean Square | F | Sig. |  |  |  |
| Between Groups | 5.234 | 2 | 2.617 | 1.441 | 0.247 |  |  |  |
| Within Groups | 83.553 | 46 | 1.816 |  |  |  |  |  |
| Total | 88.787 | 48 |  |  |  |  |  |  |
|  |  |  |  |  |  |  |  |  |
|  | *M* | *SD* |  |  |  |  |  |  |
| Man | 0.0334 | 1.38925 |  |  |  |  |  |  |
| Nonbinary | 1.1128 | 2.45302 |  |  |  |  |  |  |
| Woman | -0.2655 | 1.13657 |  |  |  |  |  |  |
|  |  |  |  |  |  |  |  |  |
| Relationship type |  |  |  |  |  |  |  |  |
|  | Sum of Squares | df | Mean Square | F | Sig. |  |  |  |
| Between Groups | 3.087 | 3 | 1.029 | 0.538 | 0.659 |  |  |  |
| Within Groups | 87.973 | 46 | 1.912 |  |  |  |  |  |
| Total | 91.06 | 49 |  |  |  |  |  |  |
|  |  |  |  |  |  |  |  |  |
|  | *M* | *SD* |  |  |  |  |  |  |
| Friend | -0.0143 | 1.31745 |  |  |  |  |  |  |
| Partner/significant other | -0.0292 | 1.8336 |  |  |  |  |  |  |
| Casual acquaintance/co-worker | -0.9267 | . |  |  |  |  |  |  |
| Other | 1.4884 | . |  |  |  |  |  |  |
|  |  |  |  |  |  |  |  |  |
| Drinking buddy status | |  |  |  |  |  |  |  |
|  | Sum of Squares | df | Mean Square | F | Sig. |  |  |  |
| Between Groups | 9.268 | 2 | 4.634 | 2.663 | 0.08 |  |  |  |
| Within Groups | 81.792 | 47 | 1.74 |  |  |  |  |  |
| Total | 91.06 | 49 |  |  |  |  |  |  |
|  |  |  |  |  |  |  |  |  |
|  | *M* | *SD* |  |  |  |  |  |  |
| No | -0.3653 | 1.18819 |  |  |  |  |  |  |
| Sometimes | 0.4312 | 1.3963 |  |  |  |  |  |  |
| Yes | 0.6964 | 1.80394 |  |  |  |  |  |  |

Note: Length of relationship was coded on a 6-point scale ranging from *0-3 months* (1) to *3 or more years* (6). Confide in each other and socialize were coded on a 4-point scale ranging from *Rarely or never* (0) to *Frequently* (3). Envision yourself drinking with this person was assessed on a 5-point scale ranging from *Definitely not* (0) to *Definitely yes* (4). Past-month frequency of drinking together and how often the network member drank alcohol were measured on an 8-point scale: ranging from *Not in the past month* (0) to *Daily* (7).
